# Medical therapy in mild autonomous cortisol secretion

**DOI:** 10.1210/jendso/bvag090

**Published:** 2026-04-09

**Authors:** Alan Kelsall, Michelle Agboola, Evie Bryden, William Healey, Katherine Tolhurst, Daniel Hind, John Newell-Price

**Affiliations:** School of Medicine and Population Health, University of Sheffield, Sheffield S10 2RX, UK; School of Medicine and Population Health, University of Sheffield, Sheffield S10 2RX, UK; School of Medicine and Population Health, University of Sheffield, Sheffield S10 2RX, UK; School of Medicine and Population Health, University of Sheffield, Sheffield S10 2RX, UK; School of Medicine and Population Health, University of Sheffield, Sheffield S10 2RX, UK; School of Health, University of Leeds, Leeds LS2 9JT, UK; School of Medicine and Population Health, University of Sheffield, Sheffield S10 2RX, UK

**Keywords:** mild autonomous cortisol secretion, medical therapy, systematic scoping review metyrapone, mifepristone

## Abstract

**Background:**

Mild autonomous cortisol secretion (MACS) has an increasing prevalence with age and is associated with cardiometabolic comorbidity and increased mortality. Current guidelines recommend the majority of patients with MACS be managed conservatively, with select people referred for laparoscopic adrenalectomy. There are currently no licensed medications for MACS.

**Objective:**

We aimed to systematically review the literature to map out the current available evidence on the use of medical therapy in the treatment of MACS and to assess the research gap. Additionally, we sought to establish the outcome measurements used, to inform the design of future prospective clinical trials.

**Design:**

We searched MEDLINE and EMBASE from conception as well as clinical trial registries to detect all original studies of medical treatments in MACS. Titles and abstracts were screened and full articles reviewed.

**Results:**

A total of 5369 results were found from the search strategy. Thirteen studies met the inclusion criteria, of which 8 were conference abstracts and 5 full-text studies were included in the final analysis. This included 2 studies of metyrapone and 3 studies that used mifepristone. Common outcome measures included blood pressure, glucose measurements, weight, and side effect data.

**Conclusion:**

There is a considerable knowledge gap with regard to the medical management of MACS. Current available evidence consists of a handful of small, heterogeneous studies with a suggestion of a potential benefit in initiating mifepristone or metyrapone in this cohort. Our results highlight the urgent need for larger, prospective studies. To support this, we have provided suggestions for outcome measures.

## Rationale

Adrenal incidentalomas (AIs) are frequently detected on performing cross-sectional imaging of the abdomen for reasons other than to assess the adrenal glands. The majority of AIs are benign adrenocortical adenomas. These adenomas are common and occur in up to 10% of the population older than 80. Approximately 20% to 50% of adrenocortical adenomas secrete excess cortisol, independently of the normal hypothalamic-pituitary regulation, termed *mild autonomous cortisol secretion* (MACS) [1]. Previously named subclinical Cushing due to biochemical evidence of corticosteroid excess but a lack of classic features associated with Cushing syndrome, MACS has been demonstrated to be associated with a significantly increased risk of cardiometabolic morbidity and mortality [2, 3]. Although several small studies have used medical therapy in MACS, there are currently no licensed medications, with the standard of care being the management of comorbidities as they arise or laparoscopic adrenalectomy in select cases following multidisciplinary team discussion [1]. We aimed to systematically review the literature to assess the effectiveness of medical interventions for MACS in improving patient outcomes.

### Objectives

This work aimed to map the extent and range of current available evidence on the effects of medical therapies directed against the hypercortisolemia associated with MACS compared to conservative management or no treatment on cardiometabolic outcomes, neuropsychiatric parameters, health-related quality of life indicators, safety, and mortality; to analyze the study designs of available evidence; and to identify current research gaps in this area.

## Methods

### Protocol and registration

A protocol was created using the Preferred Reporting Items for Systematic Reviews and Meta-Analysis Protocols (PRISMA-P) structure and revised by the authors. The final protocol was uploaded to the University of Sheffield's research data repository, ORDA, November 27, 2024 (https://doi.org/10.15131/shef.data.27917088.v1) [4].

### Eligibility

All primary research studies of medical therapeutic interventions with longitudinal data in humans with MACS were included from the selected databases since inception, with no restriction on language, so as to maximize data capture.

#### Population

The population included all adults aged 18 years or older with the presence of at least one adrenocortical adenoma and evidence of MACS as defined by a nonsuppressed serum cortisol (>50 nmol/L) following a 1-mg overnight dexamethasone suppression test (ONDST) [1].

#### Intervention

Medical therapies included but were not limited to metyrapone, mifepristone, ketoconazole, osilodrostat, relacorilant, levoketoconazole, and 11β-hydroxysteroid dehydrogenase type 1 inhibitors.

#### Outcome

Cardiometabolic parameters included blood pressure (BP), lipids, weight, glycemic control, insulin resistance, major cardiovascular events; bone mineral density and fractures; health-related quality of life; cortisol levels; neuropsychiatric measures; and mortality.

### Information sources and search

The following databases were systematically searched: MEDLINE and EMBASE. The clinical trial registries PROSPERO, WHO ICTRP, ISRCTN, EU Clinical Trials Register, and ClinicalTrials.gov were reviewed from inception. In addition, abstracts submitted to the Society for Endocrinology (UK) and European Society of Endocrinology meetings were reviewed manually via data available online (https://www.endocrine-abstracts.org/). Abstracts dating back to 2019 submitted to Endocrine Society annual meetings were reviewed via the *Journal of the Endocrine Society*. Gray literature was not included. The search strategy was created and developed alongside an experienced methodologist, and the full strategy used for MEDLINE can be found in the protocol [4]. The initial database search was performed September 6, 2024 by D.H. and A.K.

### Selection of sources of evidence

Results were uploaded to Rayyan and duplicates were removed manually by A.K. to progress to blind screening. Inclusion and exclusion criteria were prepared as documented in the protocol [4] by A.K., D.H., and J.N.P. These criteria were piloted by the reviewing team members and adjusted to optimize relevant study capture.

The results were organized alphabetically by title on Rayyan. Titles and abstracts were screened manually, individually, and blinded to other screeners. Alphabetization was used to allow efficient and transparent distribution of the workload between reviewing pairs and to ensure that each pair reviewed a distinct and nonoverlapping subset of the studies on Rayyan. The first 50% of the results were screened by K.T. and W.H. working independently and blinded to each other's decisions, with the remaining 50% screened by E.B. and M.A., again individually and blinded, with A.K. screening a proportion of results from both groups. Interrater agreement was high between pairs, demonstrating substantial agreement (K.T. and W.H. 98.3%; E.B. and M.A. 97.8%). Full-text articles from the initial relevant screening results were reviewed independently by A.K., K.T., W.H., E.V., and M.A. Where discrepancies arose, these were resolved in discussion with A.K. and J.N.P.

### Data charting process

Data charting was performed by M.A., E.B., W.H., K.T., and A.K. in discussion with J.N.P. The collected data were compared and resolution of conflicts were determined jointly and through comparison with the eligibility criteria.

### Data items

Data were collected on journal and year of publication, country of data collection, study design, number of patients, age, sex, and medical intervention.

More detailed information was collected where available on dose, frequency, and length of treatment. Data were also obtained on a range of cardiometabolic factors including BP, blood glucose, weight, lipids, quality of life, circadian rhythm, side effects, and future research recommendations.

### Synthesis of results

Studies are grouped by type of medical therapy used. Results are additionally presented by outcome data subcategory. The evidence is presented narratively alongside tabular representation.

### Risk of bias

Risk of bias assessments were not applicable for this scoping review as per PRISMA-ScR [5] and JBI guidance [6].

## Results

### Selection of sources of evidence

**Figure 1:**
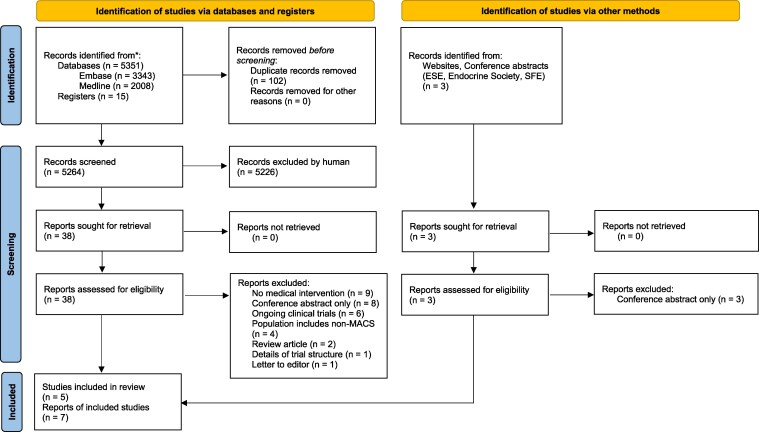
PRISMA flow diagram (Adapted from Page MJ, et al. *BMJ* 2021;372:n71. doi: 10.1136/bmj.n71. Creative Commons Attribution 4.0 International licence).

Searches of MEDLINE and EMBASE and the aforementioned clinical trial registries returned 5366 records in total (Fig. 1). Of these, 102 duplicate records were removed and 5264 titles and abstracts were reviewed manually by the authors. A total of 38 reports from the database search were further assessed for eligibility. Three reports were of the same cohort of patients published at intervals over a 4-year period and the most recent report was included, resulting in 10 studies included from database searches.

Conference abstract titles were manually screened online for the Society for Endocrinology and European Society for Endocrinology for the last 10 years and Endocrine Society for the prior 6 years using the key words from the search strategy.

Conference abstracts from both the database search and the online review without an accompanying full text were excluded from the final synthesis as they had not undergone a formal peer review process and were therefore not included in the below analyses. A full table of conference abstract results can be accessed in the supplementary material at https://doi.org/10.15131/shef.data.31821475 [7].

### Characteristics of sources of evidence

A summary of the characteristics of included studies is given in Table 1. Included articles were published between 2013 and 2025. Datasets were from 2 countries: the United Kingdom (n = 3) and United States (n = 2).

**Table 1:** Demographics of included studies

First author (publication y)	Journal	Dataset country	Abstract/full text	Study design	Intervention	No. of pats	Sex	Age (median, range), y	Adenoma	Length of treatment	Dose
Debono et al (2013) [8]	*PLOS One*	UK	Full text	Prospective	Mifepristone	6	3 M, 3 F	68.5 (58-75)	6 unilateral	4 weeks	200 mg twice a day
Debono et al (2017) [9]	*JCEM*	UK	Full text	Prospective, controlled	Metyrapone	6	3 M, 3 F	64 (59-73)	Mixture—not reported	1 day	500 mg at 6 Pm, 250 mg at 10 Pm
Ragucci et al (2017) [10]	*Case Reports in Endocrinology*	US	Full text	Case study	Mifepristone	1	F	49	Unilateral	34 weeks	300 mg increased to 900 mg per day then 600/900 mg alt day
Belokovskaya et al (2019) [11]	*Endocrine Practice*	US	Full text	Prospective	Mifepristone	8	4 M, 4 F	67 (46-82)	4 unilateral, 4 bilateral	5 pat 6 months, 3 pat 3 months	300 mg/day Increased in 2 pats to 600 mg after 2 months
Berry et al (2025) [12]	*Journal of the Endocrine Society*	UK	Full text	Retrospective, controlled	Metyrapone	15	4 M, 11 F	67 (30-85)	6 unilateral, 9 bilateral	6 months	250-500 mg at 6 Pm and 250 mg at 10 Pm

Abbreviations: F, female; *JCEM, Journal of Clinical Endocrinology and Metabolism*; M, male; pat, patient; UK, United Kingdom, US, United States.

The 5 studies were published in 5 peer-reviewed journals: *Journal of the Endocrine Society* (n = 1), *Endocrine Practice* (n = 1), *Case Reports in Endocrinology* (n = 1), *Journal of Clinical Endocrinology and Metabolism* (n = 1), and *PLOS On*e (n = 1).

### Results of individual sources of evidence

#### Synthesis of results

Across 5 studies, 2 medications were used: mifepristone (n = 3) and metyrapone (n = 2). In total, data were available for 36 people (metyrapone n = 21; mifepristone n = 15). The number of individuals per study included ranged from 1 to 15 (median 6; interquartile range [IQR] 6-8). All of the studies were full texts. One was a single patient case study. Two studies had control arms, one comparing metyrapone therapy in MACS to a group with MACS and no treatment [9] and a further retrospectively comparing metyrapone in MACS to nontreatment in an age- and sex-matched MACS group [12]. Three of the studies were prospective in design (mifepristone n = 2; metyrapone n = 1).

The total group contained 22 women and 14 men with a median age of 67 years (IQR 58.75-73.25 years). For those with data available, 17 of 30 had unilateral adenomas. The length of treatment with mifepristone ranged from 4 weeks to 34 weeks at a dose range of 300 mg total daily dose (TDD) to 900 mg/day compared to the range of reported treatment length in the metyrapone studies (1 day to 6 months) and dose (500-750 mg TDD).

### Hypertension

Four studies reported on the effects of medical therapy on hypertension (Table 2) (mifepristone n = 3; metyrapone n = 1) [8, 10-12] in a total of 30 individuals, and 2 studies on alterations to antihypertensive medication (mifepristone n = 1; metyrapone n = 1) [10, 12].

**Table 2:** Hypertension

First author (publication y)	Intervention	No. of pats	Length of treatment	Dose	Outcome
Debono et al (2013) [8]	Mifepristone	6	4 weeks	200 mg twice a day	Two pats improved their mean 24-h BP from 143/75 to 135/67 and from 138/81 to 130/81, respectively. As a group there were no significant changes in resting or ambulatory BP.
Ragucci et al (2017) [10]	Mifepristone	1	34 weeks	300 increased to 900 mg per day then 600/900 mg alt days	After 4-wk treatment, amlodipine/olmesartan medoxomil 10/20 mg discontinued. BP improved from 142/90 to 120/80 despite stopping medication.
Belokovskaya et al (2019) [11]	Mifepristone	8	5 pat 6 months, 3 pat 3 months	300 mg/day Increased in 2 pats to 600 mg after 2 months	No change in systolic (127 vs 128; *P* = .85) or diastolic BP (78 vs 77; *P* = .78)
Berry et al (2025) [12]	Metyrapone	15	6 months	250-500 mg at 6 Pm and 250 mg at 10 Pm	Compared to controls, there was a significant decrease in systolic (−17.7 vs +8.7 mm Hg; *P* = .008, n = 9) and diastolic BP (−9.9 vs +3 mm Hg; *P* = .024, n = 9) in metyrapone group. One pat in the metyrapone group required uptitration of antihypertensive medication compared to 4 in control group

Abbreviations: BP, blood pressure; pat, patient.

For 9 patients treated with metyrapone for 6 months, both systolic (−17.7 vs +8.7 mm Hg; *P* = .008) and diastolic BP (−9.9 vs +3.0 mm Hg; *P* = .024) were found to significantly improve when compared to age- and sex-matched controls, contributed to by a worsening of BP in the control group. In addition, 4 individuals in the control group had an intensification of their antihypertensive treatment, as opposed to only 1 in the intervention group [12].

In comparison, the results for mifepristone were mixed. One study of 6 patients found that 24-hour mean BP improved in 2 individuals (138/81 to 130/81 and 143/75 to 135/67, respectively) following 4 weeks of mifepristone 200 mg twice a day, but there was no statistically significant change overall as a group [8]. Similar results were seen in 8 patients treated with mifepristone for 3 to 6 months, with no significant change in systolic or diastolic BP reported (127 vs 128 mm Hg; *P* = .85; and 78 vs 77; *P* = .78; respectively) [11]. Conversely, a case study reported BP improvement from 142/90 to 120/80 following 34 weeks of mifepristone, with dose ranging between 300 and 900 mg daily, despite discontinuing amlodipine/olmesartan medoxomil at 4 weeks [10].

### Glucose control and insulin resistance

Measures of glucose control and insulin resistance were reported in 4/5 studies [8, 10-12] (Table 3) (mifepristone n = 3, metyrapone n = 1) with the most frequently used measurements being glycated hemoglobin A_1c_ (HbA_1c_) (n = 3), homeostatic model assessment of insulin resistance (HOMA-IR) (n = 2), and fasting glucose (n = 2).

**Table 3:** Glucose control and insulin resistance

First author (publication y)	Intervention	No. of pats	Length of treatment	Dose	HbA_1c_	Other
Debono et al (2013) [8]	Mifepristone	6	4 weeks	200 mg twice a day	Not reported	Reduction in lnHOMA-IR (1.0 vs 0.6; *P* = .03), lnHOMA – %β (4.8 vs 4.3; *P* = .03), lnMatsuda (1.2 vs 1.6; *P* = .03), and Geom AUC insulin 61 973 vs 40 083 pmol/L.min; *P* = .03). No improvement in fasting glucose (5.4 vs 5.6; *P* = .34) or 2 hours glucose (10.2 vs 11.4; *P* = .21). Five of 6 participants showed reduction in insulin AUC >7237 pmol/L.min, with clinically significant cardiovascular benefit demonstrated in 2 pats as defined by Helsinki Heart Study
Ragucci et al (2017) [10]	Mifepristone	1	34 weeks	300 increased to 900 mg per day then 600/900 mg alt days	Decrease from 41 to 40 mmol/mol	
Belokovskaya et al (2019) [11]	Mifepristone	8	5 pat 6 months, 3 pat 3 months	300 mg/day Increased in 2 pats to 600 mg after 2 months	No significant reduction in HbA_1c_ at 3 mo (*P* = .61) or 6 mo (*P* = .99)	Reduction in fasting glucose (*P* = .03). A significant reduction in insulin resistance as measured by HOMA-IR was observed in 6 of 8 participants in whom data were available (*P* = .03). 1 participant reduced daily insulin requirement from 127 to 85 units.
Berry et al (2025) [12]	Metyrapone	15	6 months	250-500 mg at 6 Pm and 250 mg at 10 Pm	No difference compared to control group (3.1 mmol/mol ± 4.5)	

Abbreviations: AUC, area under curve; HbA_1c_, glycated hemoglobin A_1c_; HOMA-IR, homeostatic model assessment of insulin resistance; pats, patients.

One study reported on a total of 15 patients receiving metyrapone [12]. In this group, receiving between 500 and 750 mg metyrapone over 2 doses in the evening for 6 months, there was no difference in HbA_1c_ compared to a no-treatment control group (3.1 mmol/mol ± 4.5) [12].

One case study reported a reduction in HbA_1c_ (41 to 40 mmol/mol) following 34 weeks of mifepristone therapy [10]. A prospective study of 8 patients treated with mifepristone between 3 and 6 months, however, found no significant change in HbA_1c_ at 3 months (*P* = .61), with only 3 of 8 patients found to have a decrease [11]. The same study did report a significant reduction in fasting glucose (*P* = .03) and HOMA-IR, a measure of insulin resistance, in the 6 patients for whom data were available (*P* = .03). A further prospective study of 6 patients receiving mifepristone 200 mg twice a day for 4 weeks demonstrated a reduction in InHOMA-IR (1.0 vs 0.6; *P* = .03), InMatsuda (1.2 vs 1.6; *P* = .03) and InHOMA-%β (4.8 vs 4.3; *P* = .03) and area under the curve (AUC) insulin (61 973 vs 40 083 pmol/L.min; *P* = .03), with suggested cardiovascular benefit, but no improvement in fasting glucose or 2-hour glucose following a glucose challenge [8].

### Lipids

Only 2 studies reported on lipids as an outcome measure (Table 4) (metyrapone n = 1, mifepristone n = 1) [8, 12]. In 6 patients treated with mifepristone 200 mg twice a day for 4 weeks, 2 individuals had a decrease in high-density lipoprotein (HDL) (0.84 mmol/L to 0.6 mmol/L and 0.93 mmol/L to 0.72 mmol/L, respectively); however, as a group the differences in full lipid profiles were not statistically significant [8].

**Table 4:** Lipids

First author (publication y)	Intervention	No. of pats	Length of treatment	Dose	Lipid profile
Debono et al (2013) [8]	Mifepristone	6	4 weeks	200 mg twice a day	No significant differences in full lipid profile. A decrease in HDL was found in 2 participants from 0.93 mmol/L to 0.72 mmol/L and 0.84 mmol/L to 0.6 mmol/L, respectively
Berry et al (2025) [12]	Metyrapone	15	6 months	250-500 mg at 6 Pm and 250 mg at 10 Pm	No significant difference in non-HDL compared to control group (0 vs +0.4 mmol/L)

**Abbreviation:** HDL, high-density lipoprotein.

Similarly, in 15 patients receiving evening metyrapone for 6 months, there was no significant difference in non-HDL compared to age- and sex-matched controls [12].

### Weight

Table 5 presents the results of 3 studies investigating the effect of medical therapy on weight. For mifepristone, a case study reported weight loss of 16 lb (7.3 kg) following 34 weeks of therapy [10]. In contrast, in a larger prospective study of 8 patients treated with 300 mg/day, increasing to 600 mg/day in 2 patients, there was no significant change in weight (*P* = .30), body mass index (BMI) (*P* = .46), nor waist circumference (*P* = .11), although waist circumference was noted to decrease in 6 of 8 patients [11].

**Table 5:** Weight

First author (publication y)	Intervention	No. of pats	Length of treatment	Dose	Weight loss
Ragucci et al (2017) [10]	Mifepristone	1	34 weeks	300 increased to 900 mg per day then 600/900 mg alt day	Reduction of 16 lb (7.3 kg)
Belokovskaya et al (2019) [11]	Mifepristone	8	5 pats 6 months, 3 pats 3 months	300 mg/day Increased in 2 pats to 600 mg after 2 months	No significant changes in weight (*P* = .30) or BMI (*P* = .46). In 6/8 participants, a decrease in waist circumference was reported after treatment, but this did not reach statistical significance (*P* = .11)
Berry et al (2025) [12]	Metyrapone	15	6 months	250-500 mg at 6 Pm and 250 mg at 10 Pm	No significant difference to control group (+0.2 vs −0.7 kg)

Abbreviations: BMI, body mass index; pats, patients.

One study of metyrapone measured weight as an outcome. In 15 individuals treated with evening metyrapone, there was no statistically significant difference compared with the comparator group [12].

### Circadian rhythm

A single study reviewed the effects of restoring the circadian rhythm using metyrapone in the evening at 6 Pm and 10 Pm (Table 6) [9]. AUC cortisol was shown to be elevated compared to controls between 6 and 10 Pm and 10 Pm to 2 Am prior to intervention (AUC difference 0.81 nmol/L/h; *P* = .01; and 0.86 nmol/L/h; *P* < .001; respectively) and a reduction to similar levels as controls at these time points following intervention (AUC difference −0.06 nmol/L/h; *P* = .85; and 0.10 nmol/L/h; *P* = .76) [9].

**Table 6:** Circadian rhythm

First author (publication y)	Intervention	No. of pats	Length of treatment	Dose	Circadian rhythm
Debono, et al (2017) [9]	Metyrapone	6	1 day	500 mg at 6 Pm, 250 mg at 10 Pm	24-h AUC cortisol was significantly higher in MACS participants than NFAI and healthy controls only between 6 Pm and 10 Pm (AUC difference: 0.81 nmol/L/h; *P* = .01) and between 10 Pm and 2 Am (AUC difference: 0.86 nmol/L/h; *P* < .001). Following metyrapone, there were no differences between those with MACS and controls in log-transformed AUC between 6 Pm and 10 Pm (AUC difference: −0.06 nmol/L/h; *P* = .85), between 10 Pm and 2 Am (AUC difference: 0.10 nmol/L/h; *P* = .76), nor in serum cortisol levels throughout 24-h period (*P* = .29). Salivary cortisone reflected serum results.

Abbreviations: AUC, area under curve; MACS, mild autonomous cortisol secretion; NFAI, nonfunctioning adrenal incidentaloma; pats, patients.

### Health-related quality of life

One out of the 5 studies reported on quality of life (QoL)-related outcomes. In 8 individuals treated with mifepristone for at least 3 months, no statistically significant change was reported from baseline in Cushing QoL score, Beck's Depression Scale, nor State Trait Anxiety Inventory after treatment as a group, although 6 of 7 and 5 of 7 reported improvement in Cushing QoL score and Beck's Depression Inventory, respectively (Table 7) [11].

**Table 7:** Health-related quality of life

First author (publication y)	No. of pats	Intervention	Length of treatment	Dose	Quality of life
Belokovskaya et al (2019) [11]	8	Mifepristone	5 pats 6 months, 3 pats 3 months	300 mg/day. Increased in 2 pats to 600 mg after 2 months	No significant change in QoL Becks Depression Inventory (*P* = .30) or Cushing QoL score (*P* = .27), but individual improvement in scores in 5 of 7 and 6 of 7 pats, respectively. No significant change in State Trait Anxiety Inventory (*P* = .41)

Abbreviations: pats, patients; QoL, quality of life.

### Other biochemical markers

Five studies reported on biochemical markers (mifepristone n = 3, metyrapone n = 2), with 4 pertaining to measurements of the hypothalamic-pituitary-adrenal axis (Table 8).

**Table 8:** Hypothalamic-pituitary-adrenal axis and other biochemical markers

First author (publication y)	Intervention	No. of pats	Length of treatment	Dose	HPA axis measurements	Other
Debono, et al (2013) [8]	Mifepristone	6	4 weeks	200 mg twice a day	Elevation in cortisol and ACTH in keeping with glucocorticoid receptor antagonism	No significant changes in serum osteocalcin or urine NTX/creatinine
Ragucci et al (2017) [10]	Mifepristone	1	34 weeks	300 increased to 900 mg per day then 600/900 mg alt day	Increase in ACTH from < 1 pmol/L to 2.42 at week 24	Liver function tests elevated at baseline (AST 110, NR 2-40 U/L; ALT 232, NR 2-60 U/L) and had normalized by wk 20
Belokovskaya et al (2019) [11]	Mifepristone	8	5 pats 6 months, 3 pats 3 months	300 mg/day Increased in 2 pats to 600 mg after 2 months	Increase in ACTH and DHEAS post treatment.	Increase in TSH 1.14 to 1.83 mU/L (*P* < .01). No significant decrease in potassium (4.44 vs 4.33; *P* = .77) as a group: 1 patient 5 to 2.8 mmol/L and 1 from 4.3 to 3.3 mmol/L
Debono et al (2017) [9]	Metyrapone	6	1 day	500 mg at 6 Pm, 250 mg at 10 Pm	Not reported.	IL6 elevated in MACS patients at baseline compared to controls at 4 times hourly AUCs between 10 Pm and 2 Pm (AUC difference: 0.42 pg/mL/h; *P* = .01). After intervention, IL6 was similar to control groups over all time points over 24 hours (*P* = .08).
Berry et al [12]	Metyrapone	15	6 months	250-500 mg at 6 Pm and 250 mg at 10 Pm	Following treatment, morning cortisol was 365 nmol/L (n = 13; IQR 254-431 nmol/L) and ACTH increased from 4.5 pg/mL to 7.5 pg/mL (n = 12; *P* = .032).	

Abbreviations: ACTH, adrenocorticotropin; ALT, alanine transaminase; AST, aspartate transaminase; AUC, area under curve; DHEAS, dehydroepiandrosterone sulfate; IL6, interleukin 6; IQR, interquartile range; MACS, mild autonomous cortisol secretion; NR, normal range; pats, patients; TSH, thyrotropin.

Three studies of mifepristone, including 15 individuals, demonstrated an increase of adrenocorticotropin (ACTH) in keeping with glucocorticoid receptor antagonism [8, 10, 11] and one reported an increase in thyrotropin (1.14 to 1.83 mU/L; *P* < .01) but no statistically significant change in potassium (4.44 vs 4.33 mmol/L; *P* = .77) as a group, although 2 patients had a reduction in potassium greater than or equal to 1 mmol/L [11].

No significant differences were observed in serum osteocalcin or urine NTX/creatinine before and after therapy in 6 patients treated with mifepristone 200 mg twice a day for 4 weeks [8].

A further case study of a patient with nonalcoholic fatty liver disease commenced on mifepristone found that liver function tests aspartate transaminase (AST) and alanine transaminase (ALT) normalized after 20 weeks of therapy [10].

With regard to metyrapone, 13 patients treated with an evening dose had a normal morning cortisol (365 nmol/L, IQR 254-431 nmol/L) with the authors suggesting the safety of its use [12].

Additionally, normalization of interleukin-6 to levels in MACS patients similar to that of controls was demonstrated following the restoration of the circadian rhythm in those on metyrapone intervention [9].

### Adverse events

Four of the studies reported on the presence or absence of adverse events (Table 9) (metyrapone n = 2, mifepristone n = 2) for a total of 35 patients (metyrapone n = 21, mifepristone n = 14). The majority of studies provided limited information and did not directly report the medication as the cause of the adverse event, nor graded the severity of the events. We have grouped events descriptively based on the original authors’ reports.

**Table 9:** Adverse events

First author (publication y)	Intervention	No. of pats	Length of treatment	Dose	Adverse events
Debono et al (2013) [8]	Mifepristone	6	4 weeks	200 mg twice a day	Two participants developed symptoms of lassitude, fatigue, and hypokalemia (3.2 mmol/L and 3.1 mmol/L) at end of week 2 and end of week 4, respectively
Belokovskaya et al (2019) [11]	Mifepristone	8	5 pats 6 months, 3 pats 3 months	300 mg/day Increased in 2 pats to 600 mg after 2 months	3/8 withdrew after 3 months. 1 participant reported fatigue, 1 hypokalemia, and 1 symptoms of cortisol withdrawal but no evidence of adrenal insufficiency
Debono et al (2017) [9]	Metyrapone	6	1 d	500 mg at 6 Pm, 250 mg at 10 Pm	6 adverse events in 4 pats: 4 mild headaches (1 possibly related), 1 episode of mild dizziness, and 1 episode of hypertension
Berry et al (2025) [12]	Metyrapone	15	6 months	250-500 mg at 6 Pm and 250 mg at 10 Pm	Well tolerated by 10/15. Dizziness (n = 3), nausea (n = 2), headaches (n = 1). Stopped in 2/15: 1 female developed asymptomatic high androgens and 1 participant had diarrhea. No adrenal crises

**Abbreviation:** pats, patients.

A total of 9 of 21 patients taking metyrapone had 14 adverse events, occurring in those prescribed a TDD range of 500 to 750 mg metyrapone. The most commonly reported adverse events were mild headache (n = 5) [9, 12], dizziness (n = 4) [9, 12], nausea (n = 2) [12], diarrhea (n = 1) [12], hypertension (n = 1) [9], and elevated androgens (n = 1) [12]. Of these, diarrhea and elevated androgens were attributed to the effects of metyrapone by the original authors [12], and one event of headache was described as possibly related [9].

From the pooled mifepristone data, a total of 7 adverse events were reported in 5 of 14 patients, with the most common being hypokalemia (n = 3) [8, 11], fatigue (n = 3) [8, 11], and cortisol withdrawal symptoms without evidence of adrenal insufficiency (n = 1) [11].

### Future recommendations

In all 5 studies (Table 10), the authors gave research recommendations, with the predominant theme that further large prospective trials were required to assess the clinical effects of metyrapone and mifepristone on metabolic, cardiovascular, bone, and neuropsychiatric markers [8-12].

**Table 10:** Future recommendations

First author (publication y)	Intervention	No. of pats	Length of treatment	Dose	Research recommendations
Debono, et al (2013) [8]	Mifepristone	6	4 weeks	200 mg twice a day	Randomized controlled studies required to further establish whether GR blockade or cortisol-lowering strategies offer an alternative to surgical intervention
Ragucci et al (2017) [10]	Mifepristone	1	34 weeks	300 increased to 900 mg per d then 600/900 mg alt day	Larger prospective studies needed
Belokovskaya et al (2019) [11]	Mifepristone	8	5 pats 6 months, 3 pats 3 months	300 mg/day. Increased in 2 pats to 600 mg after 2 months	Further studies required to determine effect of steroidogenesis inhibitors and GR antagonists on mood, cardiovascular, metabolic, and bone parameters in patients with MACS
Debono et al (2017) [9]	Metyrapone	6	1 day	500 mg at 6 Pm, 250 mg at 10 Pm	A randomized, controlled trial is needed in a larger cohort of MACS patients to further assess effect of metyrapone on clinical outcomes
Berry et al (2025) [12]	Metyrapone	15	6 months	250-500 mg at 6 Pm and 250 mg at 10 Pm	A larger prospective study is needed to further investigate the metabolic outcomes of metyrapone in MACS

Abbreviations: GR, glucocorticoid receptor; MACS, mild autonomous cortisol secretion; pats, patients.

### Other studies of interest

Three prospective studies investigated medical therapies in people with hypercortisolism with cohorts that included patients with MACS; however, as the studies did not subgroup MACS as per aforementioned our inclusion criteria and the results for MACS patients were not clearly delineated, they have not been included with those listed earlier. We feel that a descriptive analysis of these studies does have merit with regard to highlighting the outcome measures used and adverse events, in the design of future prospective trials.

Oda et al [13] investigated the efficacy of an 11β-HSD1 inhibitor, S-707106, in an open-label prospective study in 15 patients with Cushing syndrome (Cushing disease n = 2, ectopic ACTH syndrome n = 1, adrenal Cushing n = 1) and MACS (n = 11) and with a history of impaired glucose tolerance. Participants either had a residual tumor or were not suitable for surgical intervention. The initial dose was 200 mg daily, increased to 200 mg twice daily if no improvement in glucose parameters was found at 12 weeks. The primary end point of a 25% reduction from baseline in AUC plasma glucose following oral glucose tolerance test at 24 weeks was not met, with a reduction of 7.1% (*P* = .033) and 2.7% (*P* = .18) reported at 12 and 24 weeks. A greater percentage change was noted in those with a BMI greater than 30. Fasting plasma glucose and HbA_1c_ were similar following treatment; however, there was a significant reduction in body fat percentage (−2.5%; *P* < .001) and weight (61.42 vs 60.78 kg) with an increase in body muscle percentage (2.4%; *P* < .001) and a reduction in HOMA-IR at 24 weeks (−0.91 ± 0.82; *P* = .0011). With regard to other biomarkers, no change was found in morning serum cortisol. Dehydroepiandrosterone sulfate was found to be elevated in 13 patients, but with no clinical signs of androgen excess. Both ALT and ALT/AST were significantly reduced following treatment, as well as γ-glutamyl transferase and HDL cholesterol. Across 15 participants, 60 adverse events occurred with the most common being gastrointestinal side effects (n = 10), disorders of the skin (n = 10), infections (n = 9), and fatigue (n = 7). Two severe adverse events were reported (pyelonephritis n = 1; urinary tract infection n = 1), with both thought to be related to Cushing syndrome, and the 11β-HSD1 inhibitor was not discontinued.

The recently published CATALYST study [14] randomly assigned 136 adults with poorly controlled type 2 diabetes despite medication, and an ONDST cortisol greater than 50 nmol/L to receive either mifepristone (n = 91; dose range 300-900 mg once daily) or placebo (n = 45). Randomization was stratified by the presence or absence of adrenal abnormalities on abdominal computed tomography scan, which were present in 27.9%. A significant reduction in HbA_1c_ was reported at 24 weeks in the mifepristone compared to placebo group (−1.32%; 95% CI, −1.81% to −0.83%), with similar results being found for those with adrenal abnormalities (−1.41%; 95% CI, −2.34% to −0.48%). Common side effects of mifepristone included hypokalemia, fatigue, and nausea occurring in 29.7%, 20.9%, and 20.9% of participants, respectively, but with no reported cases of adrenal insufficiency.

On review of the supplementary material, adrenal abnormalities included nodule, enlargement, or other abnormality with no provision of results or statistical analyses specifically for the adrenal nodule group, and therefore the aforementioned results have not been collated with those of the other included studies.

Musolino et al [15] reported on 20 patients with mild hypercortisolism (defined as cortisol >50 nmol/L following ONDST, urine free cortisol <1.5× upper limit of normal, 1 or more cortisol-related comorbidities and <2 Cushing'specific features) prospectively prescribed either metyrapone 250 mg at 10 Pm (n = 8) for 24 weeks or metyrapone 250 mg at 10 Pm for 12 weeks followed by the addition of 250 mg at 1 Pm (n = 12). Imaging and biochemistry confirmed either a pituitary (n = 2) or adrenal (n = 18) source of hypercortisolism. Average 24-hour systolic BP improved by 5 mm Hg or more in 40% after 24 weeks (*P* = .008), with an improvement in diastolic BP reported in 30% at 12 weeks but only 20% at 24 weeks (*P* = .125). There was no statistically significant improvement in HbA_1c_ in those with (7.2 vs 6.8; *P* = .25) or without type 2 diabetes (6.3 vs 6.1; *P* = .09) following treatment. There was no significant difference reported in salivary cortisol AUC over a 24-hour period or when split into intervals. No side effects were reported and there was no evidence of adrenal insufficiency.

## Discussion

### Summary of evidence

Through this scoping review we have comprehensively assessed the use of medical therapy in MACS, study designs, and the range of cardiometabolic and other outcome measures used so as to inform future trials. To our knowledge, this is the first review to systematically assess the breadth of literature on medical interventions in MACS. The key finding, as evidenced earlier, is that there is a substantial knowledge gap, with the majority of the included articles being small studies, largely uncontrolled and heterogeneous both in design and populations. In addition, multiple different outcome measures were assessed across studies, making meta-analysis of results difficult and limiting the ability to provide strong clinical recommendations, which is reflected in current guidelines [1].

From the limited available evidence, both metyrapone and mifepristone were well tolerated and demonstrated cardiometabolic benefit in MACS across a range of ages. Metyrapone demonstrated a reduction in hypertension but less of an effect on glucose measures and insulin resistance, whereas mifepristone showed some improvement of insulin resistance but mixed results with regard to hypertension. Neither medication significantly reduced lipids. Limited data reported the safe use of mifepristone in nonalcoholic fatty liver disease (n = 1) [10].

To date, there have been no randomized controlled trials comparing the medical treatment of MACS with placebo or with surgical intervention. Current European guidelines recommend that the options for management consist of two strands: monitoring for cortisol-related comorbidities, or surgery in selected cases [1].

In clinical practice, the minority of individuals with AIs detected on cross-sectional imaging are referred for endocrine assessment. Of those that are, the majority are discharged to the community with a recommendation for annual monitoring of comorbidities. The true burden of MACS is therefore likely to be underappreciated, with arising cortisol-related comorbidities often not linked to the underlying cause.

The recent CHIRACIC study prospectively investigated 52 people with MACS, demonstrating a significant reduction in the number of antihypertensive medications prescribed in those randomly assigned to adrenalectomy compared to a conservatively managed control group (46% vs 15%; adjusted risk difference 0.34; 95% CI, 0.11-0.58). The majority of those in the adrenalectomy group were able to stop antihypertensive treatment completely (52%) compared to none in the control group. These data suggest the causal association of MACS and hypertension and reinforce the benefits of active intervention. Concordant data from Koh et al [16] found improvements in BP control, glucose, and weight in 46 patients with MACS randomly assigned to adrenalectomy compared to an age- and sex-matched control group. Further, a detrimental effect of conservative management was highlighted with a greater likelihood of deterioration in cardiometabolic parameters from baseline in the control group after a median of 48 months follow-up. Further data from a multicenter randomized controlled trial of 43 patients with MACS demonstrated normalization of ONDST serum cortisol after 2 years following adrenalectomy in 19 of 21 patients compared to 2 of 22 in the control group who were conservatively managed [17]. This was associated with a reduction in office BP of 10 mm Hg or more and daily defined doses of antihypertensive in 9 of 12 compared with 4 of 15 in the control group (*P* = .01), although no difference was noted in mean 24-hour BP before and after adrenalectomy nor compared to the control group.

A mid-2023 estimate of UK population size was 68 265 209 with 9 434 457 older than 70 years [18]. If 10% of this group are estimated to have an adrenal adenoma, with 20% to 50% of these tumors secreting excess cortisol, then approximately 188 689 to 471 723 people in the United Kingdom have MACS. This of course would be an underestimate as it does not take into account those younger than 70 with MACS. Prevalence will likely continue to increase in the near future, with an aging population. These large numbers illustrate that surgical intervention for all appropriate patients with MACS will never be possible at the population level and challenging in a cohort of patients at higher risk of surgery due to frailty and other comorbidities, before even taking into account patient choice.

Conservative management may also be suboptimal. Managing MACS-associated comorbidities, such as hypertension and diabetes, as they arise does not tackle the root cause, with the potential to lead to increased burden on community physicians as well as side effects associated with polypharmacy. Additionally, some features related to cortisol excess, such as cognitive impairment [19], cannot currently be managed effectively through medication that addresses comorbidities such as hypertension and impaired glucose homeostasis.

There is, therefore, a substantial need for robust and well-structured prospective clinical trials to assess current and new medications for MACS, and a number of clinical trials are ongoing as documented in Table 11.

**Table 11:** Ongoing clinical trials

Trial ID	Title	Intervention	Recruitment status	Primary end point
EUCTR2022-000161-40-AT	Investigating cardiometabolic risk factors and changes in chronobiology patterns in patients with autonomous adrenal cortisol secretion	Metyrapone	Active, not recruiting	Hepatic lipid content on MR spectroscopy
ISRCTN42727091	Dissecting the Contribution of glucocorticoid metabolism in mild autonomous cortisol secretion: a randomized controlled trial of the 11β-HSD1 inhibitor SPI-62	SPI-62	Recruiting	Change in glucose disposal
NCT06801249	Effect of metyrapone on cardiovascular risk factors in patients with adrenal incidentalomas and Cushing’s syndrome	Metyrapone	Recruiting	Change in number and/or dosage of antihypertensives following 12 mo of metyrapone treatment
EUCT2024-514143-28-00	Diagnostic treatment-trial for autonomous cortisol secretion—a tool to select patients for adrenalectomy (Metycor)	Metyrapone	Recruiting	Change in blood pressure, glucose metabolism, and cortisol levels
NCT07138274	A phase II, randomized, double-blind, placebo-controlled study evaluating the safety and efficacy of metyrapone in subjects diagnosed with mild autonomous cortisol secretion (MACS)	Metyrapone	Not yet recruiting	Change in glucocorticoid to androgen ratio. Waking and bedtime salivary cortisone (δ salivary cortisone).
NCT07104812	Impact of 1 mg osilodrostat therapy on mild autonomous cortisol secretion (MACS)	Osilodrostat	Not yet recruiting	Adverse events. Proportion that experience adrenal insufficiency

Abbreviation: MR, magnetic resonance.

We suggest that future trials should be structured to compare medical therapy against placebo or surgery in randomized controlled trials. Ideally there would be direct comparison against the currently recommended strategy of aggressive management of comorbidities as they arise, although this would be difficult to capture in the time period of most funded clinical trials. Suggested outcome measures would include systolic and diastolic BP, blood glucose measurements including continuous glucose monitoring, HbA_1c_, and assessment of insulin sensitivity; lipids, waist circumference, height, and weight; cognitive screening, anxiety and depression questionnaires, and for therapies that lower circulating cortisol, early-morning and late-night serum or salivary cortisol/cortisone samples and safety data.

There are several limitations to this scoping review. Included articles were not formally graded for risk of bias as per current guidelines on scoping reviews; however, the majority of included studies were of low-quality evidence, with small sample sizes and therefore prone to bias. In addition the small number of heterogeneous studies does not allow for meta-analysis of the data, and it may be that in larger datasets, similar results would not be observed. The strengths include a systematic approach to the review of all available literature on the included databases.

## Conclusion

The current available evidence for medical treatment in MACS is sparse, heterogeneous, and predominantly consists of low-quality evidence. Both metyrapone and mifepristone have been used in the medical treatment of MACS in a number of small studies with improvement in various cardiometabolic parameters reported; however, there is insufficient evidence to provide robust clinical recommendations. This systematic scoping review maps the current literature and demonstrates the urgent necessity for future large prospective trials in the area of medical therapy in this common condition.

## Data Availability

Data sharing is not applicable to this article as no datasets were generated or analyzed during the current study.

## Abbreviations

ACTH: adrenocorticotropic hormone
AI: adrenal incidentaloma
ALT: alanine transaminase
AST: aspartate transaminase
AUC: area under curve
BMI: body mass index
BP: blood pressure
HbA_1c_: glycated hemoglobin A_1c_
HDL: high-density lipoprotein
HOMA-IR: homeostatic model assessment of insulin resistance
IQR: interquartile range
MACS: mild autonomous cortisol secretion
ONDST: overnight dexamethasone suppression test
QoL: quality of life
TDD: total daily dose

## References

[1] Fassnacht M, Tsagarakis S, Terzolo M, et al European Society of Endocrinology clinical practice guidelines on the management of adrenal incidentalomas, in collaboration with the European Network for the Study of Adrenal Tumors. Eur J Endocrinol. 2023;189(1):G1‐G42. 37318239 10.1093/ejendo/lvad066

[2] Pelsma ICM, Fassnacht M, Tsagarakis S, et al Comorbidities in mild autonomous cortisol secretion and the effect of treatment: systematic review and meta-analysis. Eur J Endocrinol. 2023;189(4):S88‐S101. 37801655 10.1093/ejendo/lvad134

[3] Deutschbein T, Reimondo G, Di Dalmazi G, et al Age-dependent and sex-dependent disparity in mortality in patients with adrenal incidentalomas and autonomous cortisol secretion: an international, retrospective, cohort study. Lancet Diabetes Endocrinol. 2022;10(7):499‐508. 35533704 10.1016/S2213-8587(22)00100-0 PMC9679334

[4] Kelsall A, Hind D, Newell-Price J. Protocol for Systematic Scoping Review Medical Interventions for Mild Autonomous Cortisol Excess (MACS). The University of Sheffield; 2024.

[5] Tricco AC, Lillie E, Zarin W, et al PRISMA extension for scoping reviews (PRISMA-ScR): checklist and explanation. Ann Intern Med. 2018;169(7):467‐473. 30178033 10.7326/M18-0850

[6] Peters MDJ, Marnie C, Tricco AC, et al Updated methodological guidance for the conduct of scoping reviews. JBI Evid Synth. 2020;18(10):2119‐2126. 33038124 10.11124/JBIES-20-00167

[7] Kelsall A, Newell-Price J. Sheffield Online Research Data. The University of Sheffield; March 2026. 10.15131/shef.data.31821475

[8] Debono M, Chadarevian R, Eastell R, Ross RJ, Newell-Price J. Mifepristone reduces insulin resistance in patient volunteers with adrenal incidentalomas that secrete low levels of cortisol: a pilot study. PLoS One. 2013;8(4):e60984. 23577182 10.1371/journal.pone.0060984 PMC3618218

[9] Debono M, Harrison RF, Chadarevian R, Gueroult C, Abitbol JL, Newell-Price J. Resetting the abnormal circadian cortisol rhythm in adrenal incidentaloma patients with mild autonomous cortisol secretion. J Clin Endocrinol Metab. 2017;102(9):3461‐3469. 28911138 10.1210/jc.2017-00823 PMC5587065

[10] Ragucci E, Nguyen D, Lamerson M, Moraitis AG. Effects of mifepristone on nonalcoholic fatty liver disease in a patient with a cortisol-secreting adrenal adenoma. Case Rep Endocrinol. 2017;2017(1):6161348. 29348947 10.1155/2017/6161348 PMC5733994

[11] Belokovskaya R, Ravikumar A, Arumugam D, et al Mifepristone treatment for mild autonomous cortisol secretion due to adrenal adenomas: a pilot study. Endocr Pract. 2019;25(8):846‐853. 31070948 10.4158/EP-2019-0047 PMC9125788

[12] Berry S, Iqbal A, Newell-Price J, Debono M. Efficacy and tolerability of metyrapone in mild autonomous cortisol secretion: real-world findings from clinical practice. Clin Endocrinol (Oxf). 2025;104(3):215‐221. 41198609 10.1111/cen.70056 PMC12865743

[13] Oda S, Ashida K, Uchiyama M, et al An open-label phase I/IIa clinical trial of 11β-HSD1 inhibitor for Cushing's syndrome and autonomous cortisol secretion. J Clin Endocrinol Metab. 2021;106(10):e3865‐e3880. 34143883 10.1210/clinem/dgab450

[14] DeFronzo RA, Fonseca V, Aroda VR, et al Inadequately controlled type 2 diabetes and hypercortisolism: improved glycemia with mifepristone treatment. Diabetes Care. 2025;48(12):2036‐2044. 40550011 10.2337/dc25-1055 PMC12635952

[15] Musolino A, Favero V, Parazzoli C, et al Effects of metyrapone in patients with mild hypercortisolism. Eur J Endocrinol. 2025;193(5):644‐653. 41102583 10.1093/ejendo/lvaf216

[16] Koh JM, Song K, Kwak MK, et al Adrenalectomy improves body weight, glucose, and blood pressure control in patients with mild autonomous cortisol secretion: results of an randomized controlled trial by the co-work of adrenal research (COAR) study. Ann Surg. 2024;279(6):945‐952. 38126763 10.1097/SLA.0000000000006183

[17] Ueland GÅ, Ragnarsson O, Heie A, et al Randomized trial studying metabolic outcomes and quality of life after adrenalectomy versus conservative management for mild autonomous cortisol secretion. Endocr Connect. 2025;14(7):e250361. 40600855 10.1530/EC-25-0361 PMC12281625

[18] Office for National Statistics (ONS) . ONS website, statistical bulletin, Population estimates for the UK, England, Wales, Scotland, and Northern Ireland: mid-2023. Released 8 October 2024. Accessed 10 June 2025. https://www.ons.gov.uk/peoplepopulationandcommunity/populationandmigration/populationestimates/bulletins/annualmidyearpopulationestimates/mid2023

[19] Saini J, Nathani R, Singh S, et al Cognition in patients with mild autonomous cortisol secretion at baseline and post-adrenalectomy. Eur J Endocrinol. 2024;191(6):636‐645. 39671564 10.1093/ejendo/lvae157 PMC11662234

